# The carbon footprint of breastmilk substitutes in comparison with breastfeeding

**DOI:** 10.1016/j.jclepro.2019.03.043

**Published:** 2019-06-10

**Authors:** Johan O. Karlsson, Tara Garnett, Nigel C. Rollins, Elin Röös

**Affiliations:** a Department of Energy and Technology, Swedish University of Agricultural Sciences, Uppsala, Sweden; b Food Climate Research Network, Environmental Change Institute, Oxford University, Oxford, UK; c Department of Maternal, Newborn, Child and Adolescent Health (MCA), WHO, Geneva, Switzerland

**Keywords:** Climate impact, Infant formula, LCA, Lifecycle assessment, Uncertainty, Sensitivity, CFP, Carbon footprint, BMS, Breastmilk substitute, LUC, Land use change

## Abstract

Breastfeeding is one of the foundations of child health, development and survival. Breastmilk substitutes (BMS) are associated with negative influences on breastfeeding practices and subsequent health concerns and, as with all foods, production and consumption of BMS comes with an environmental cost. The carbon footprint (CFP) of production and consumption of BMS was estimated in this study. To illustrate regional differences among the largest producers and consumers, the CFP of BMS production in New Zealand, United States (USA), Brazil and France and the CFP of BMS consumption in United Kingdom (UK), China, Brazil and Vietnam were assessed. The CFP values were then compared with the CFP of breastfeeding arising from production of the additional food needed for breastfeeding mothers to maintain energy balance (approximately 500 kcal per day). The CFP of production was estimated to be 9.2 ± 1.4, 7.0 ± 1.0, 11 ± 2 and 8.4 ± 1.3 kg CO_2_e per kg BMS in New Zealand, USA, Brazil and France, respectively, with the largest contribution (68–82% of the total) coming from production of raw milk. The CFP of consumption, which included BMS production, emissions from transport, production and in-home sterilisation of bottles, and preparation of BMS, was estimated to be 11 ± 1, 14 ± 2, 14 ± 2 and 11 ± 1 kg CO_2_e per kg BMS in UK, China, Brazil and Vietnam, respectively. Comparison of breastfeeding with feeding BMS showed a lower CFP from breastfeeding in all countries studied. However, the results were sensitive to the method used to allocate emissions from raw milk production on different dairy processing co-products (i.e. BMS, cream, cheese and lactose). Using alternative allocation methods still resulted in lower CFP from breastfeeding, but only slightly for UK, Brazil and Vietnam. Care is also needed when interpreting findings about products that are functionally different as regards child health and development.

## Highlights

• Feeding breastmilk substitutes has a larger carbon footprint than breastfeeding.
• Dairy farming contributes most to the carbon footprint of breastmilk substitutes.
• Sterilisation of bottles contributes most to consumption phase emissions.
• The results are sensitive to the allocation method used for dairy co-products.

## 1 Introduction

Breastfeeding is one of the foundations of child health, development and survival, especially in areas where diarrhoea, pneumonia and undernourishment are common causes of mortality among children younger than five years (Victora et al., 2016). Increasing breastfeeding duration has been associated with reduced risk of childhood infection, better mental health (Oddy et al., 2010) and probably reduced risk of non-communicable diseases, including overweight and diabetes (Kelishadi and Farajian, 2014). Breastfeeding has also been reported to have significant health benefits for women, providing protection against various cancers and diabetes (Blincoe, 2005). Some of the positive health effects of breastfeeding has however recently been criticized and linked to positive maternal selection in previous studies (i.e. mothers who choose to breastfeed are generally more informed about infant health and nutrition, which might explain part of the link between breastfeeding and positive child health outcomes). By controlling for ‘intention to breastfeed’ Raissian and Su (2018) show that expectant mothers that intended to breastfeed had healthier infants irrespective of whether they actually ended up breastfeeding or not. Nevertheless, the World Health Organization (WHO) recommends exclusive breastfeeding for the first six months of life, followed by continued breastfeeding with appropriate complementary foods for up to two years or beyond.^1^ Most (although not all) women are physically able to breastfeed, but a wide range of historical, cultural and socio-economic factors affect the choice to initiate and continue breastfeeding. One factor with a strong influence on uptake and continuation of breastfeeding is the marketing and availability of infant formula or breastmilk substitutes (BMS) (Piwoz and Huffman, 2015), which are products intended to supplement or replace breastmilk in infant feeding. Sales of BMS have increased by approximately 8% year-on-year globally, even during the economic recession and in almost all countries and regions (Rollins et al., 2016).

Breastmilk substitutes can be subdivided into four main categories based on intended use: i) Standard infant milk formula, for use from birth until 6 months; ii) follow-on milk formula, for babies aged 6–12 months; iii) toddler milk formula or growing up milk, for children older than 12 months; and iv) special baby milk formula, which includes several types of specialised formula intended for babies with special nutritional needs or allergies, e.g. soy-based formula, and represents only a small proportion of BMS sales (Euromonitor International, 2015). Most BMS are based on bovine milk, which is further processed to resemble human milk (Martin et al., 2016). The milk is skimmed to reduce the saturated fat content and, since human milk has a larger fraction of whey proteins than cow's milk, additional whey proteins are added to obtain a comparable protein composition. Lactose or glucose syrup is added to increase the carbohydrate content, a blend of vegetable oils to provide unsaturated fatty acids (GEA, 2010) and a mix of vitamins and minerals to satisfy nutritional requirements. Various standards, such as the Joint FAO/WHO Codex Alimentarius (2007) and the US Food and Drug Administration (2018), regulate the appropriate amount of protein, carbohydrates, fat, minerals and vitamins in BMS.

Production and consumption of BMS, as with all foods, comes with an environmental cost. The global food system, from production through all stages of processing, distribution, food preparation and consumption, accounts for an estimated 19–29% of global anthropogenic greenhouse gas emissions (Vermeulen et al., 2012). Animal-based food products generally have a higher climate impact than plant-based foods (Clune et al., 2017; Poore and Nemecek, 2018), due to emissions from feed production, manure management and, in the case of ruminant animals, enteric fermentation. Life cycle assessment has been used to estimate the environmental impact, and particularly the climate impact, of many food products (see Clune et al. (2017) for a review). The climate impact of BMS has received some attention (Dadhich et al., 2015), but this topic has to our knowledge not been covered in the peer-reviewed literature and no previous studies have compared the climate impact of BMS to that of breastfeeding.

The aim of this study was therefore to estimate the greenhouse gas emissions, or carbon footprint (CFP), from production and consumption of BMS. To illustrate regional differences, we calculated the CFP from production of BMS in four case countries and from consumption of BMS in four case countries. Finally, we compared the CFP from using BMS with that of breastfeeding. We limited our assessment to BMS targeted at infants aged 0–6 months, since the WHO recommends exclusive breastfeeding during this period and it is thus the category of BMS that most directly competes with breastfeeding. All BMS are sold in powder, liquid concentrate or liquid ready-to-feed form. We limited our assessment to the powdered form, which constitutes 90% of the global market for BMS intended for infants younger than 6 months (Euromonitor International, 2015). For simplicity, we hereafter use the term BMS synonymously with this specific subcategory.

## 2 Materials and methods

### 2.1 Life cycle assessment and carbon footprint

Life cycle assessment (LCA) is a well-established standardised method to assess the environmental impacts from a product or service (ISO, 2006a,b). Use of natural resources, including energy, land, minerals and metals, and outputs in the form of products, by-products, emissions and waste are quantified for all steps in the life cycle of the product under study and impacts are aggregated into different environmental impact categories and related to the ‘functional unit’. In the case of food the ‘functional unit’ is typically 1 kg of a certain food product on the plate or at the farm or retail gate. LCA has been carried out on a multitude of food products and production systems (Clark and Tilman, 2017; Poore and Nemecek, 2018) and although the field is young it is becoming increasingly established being used extensively in both research and in the food industry. However, several methodological challenges exist in LCA on food including e.g. how to handle uncertainty and variability in the inventory modelling, how to account for the function of different foods within the functional unit and how LCA results are best translated to policy or consumer guidance (Gava et al., 2018; Notarnicola et al., 2017). With the rising urgency of climate change, an increased focus has been put on the climate impact of food specifically. A ‘carbon footprint’ is a subset of a full LCA in which only the climate impact is assessed. Due to the large interest in this indicator, ISO have issued a standard that deals specifically with the carbon footprint of products, the ISO 14067 (ISO, 2013).

This study was performed and documented (in this article and its Annexes) according to the ISO 14067 standard with the following exception: we did not estimate biogenic carbon uptake (i.e. the carbon temporarily stored in the BMS), as this is not commonly included in the CFP of food products (Clune et al., 2017) and can cause confusion. For the impact assessment the Global Warming Potential over a one hundred year time frame (GWP_100_) factors from IPCC AR4 (Forster et al., 2007) were used, as the underlying data also used these factors. The sensitivity of the results to using the IPCC AR5 (Myhre et al., 2013) factors was tested. A full LCA was not performed due to lack of data on other impact categories. We acknowledge the need to complement our results on the climate impact of BMS versus breastfeeding with studies on other environmental impacts in order to draw conclusion on the full environmental impacts of the two. However, Poore and Nemecek (2018) found that the climate impact of many animal products is positively correlated with both acidifying and eutrophying emissions why the climate impact of a food product may in some cases serve as a proxy also for other indicators revealing more generally a products environmental profile.

### 2.2 Study set-up

**Fig. 1:**
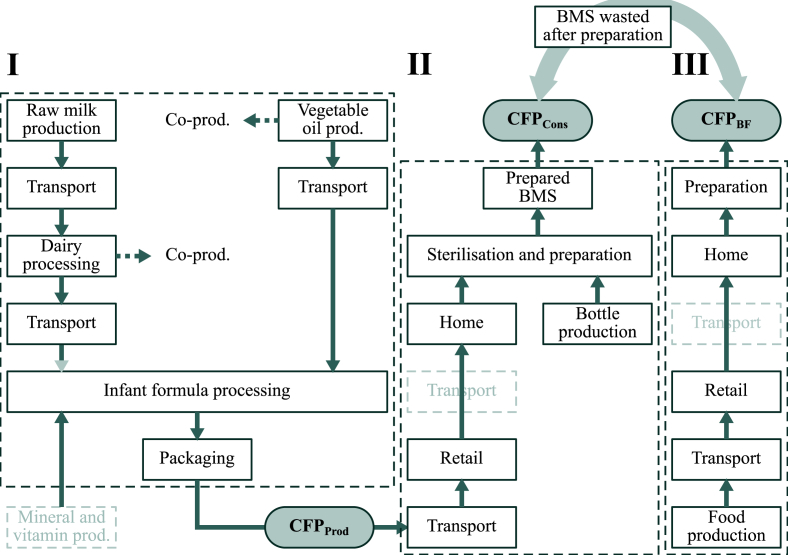
The three steps followed in this study when assessing the carbon footprint (CFP) of breastmilk substitutes (BMS), including system boundaries (hatched lines) and the location of the estimated CFP. Shaded boxes indicate processes not included in the assessment. BMS wasted after preparation was accounted for in the CFP of breastfeeding to estimate the amount of breastfeeding equivalent to 1 kg powdered BMS.

This study was undertaken in three stages (Fig. 1). First, a partial CFP from *producing and packaging* 1 kg *BMS* (CFP_Prod_) was estimated (Section 2.5.1-5) for the four production case countries (Section 2.4), including all stages in the life cycle up until the BMS leaves the factory gate. Next, the CFP from *consuming* 1 kg *BMS* (CFP_Cons_) in the four consumption case countries was calculated (Section 2.5.6). The consumption CFP was the sum of emissions from the production stage, to which were added emissions related to transport from production site to retail outlet, production and sterilisation of baby bottles and heating water to prepare the BMS. Finally, we estimated the CFP from *breastfeeding the equivalent of* 1 kg *BMS* (CFP_BF_) by accounting for the additional food needed for breastfeeding mothers (Section 2.6).

Data on the CFP of the main ingredients of BMS (raw milk and vegetable oils) and emissions from the production of materials for packaging and feeding bottles and for transport were taken from the literature (Section 2.5, 2.6, 2.7). As a quality check, our estimated inventory data were validated against confidential data on BMS production provided by the dairy cooperative Arla foods.

### 2.3 Functional unit

The functional unit was selected as the equivalent of 1 kg of BMS powder with an energy content of 21.7 kJ/g. This functional unit implied that calculation of the CFP of breastfeeding also had to account for BMS wasted after preparation, as this affects the amount of breastfeeding that is equivalent to 1 kg BMS (indicated in Fig. 1). The reason for selecting this functional unit rather than e.g. ‘providing adequate feeding for an infant from birth up until 6 months of age’ was to facilitate easier interpretation and usability of results in future studies; a CFP on the basis of ‘mass of product’ was considered preferable to a CFP on the basis of a more complex function.

### 2.4 Selection of case countries

The four production case countries (New Zealand, United States [USA], Brazil, France) were selected based on production volume of milk powder. Together, they accounted for some 49% of global milk powder production during 2010–2014.^2^ The consumption case countries (United Kingdom [UK], China, Brazil, Vietnam) were selected based on data from a market assessment of the BMS industry (Euromonitor International, 2015). The selection was made to include countries where BMS is a growing or already large market, both in total and per capita terms, and to include a geographical and socio-economic spread of contexts. China is the largest market for BMS, with a retail volume in 2014 reaching 177,000 tonnes. The United Kingdom has the largest retail volume per capita, Vietnam shows the greatest retail growth, with a doubling in sales between 2010 and 2014, and Brazil is the seventh largest market for BMS, with a growth rate of almost 50% between 2010 and 2014. We coupled each consumption case country with one of the production case countries and assumed that BMS consumed in UK, China, Brazil and Vietnam was produced in France, New Zealand, Brazil and France, respectively. This coupling was based on trade data^3^ for the period 2012–2016 and represents a plausible trade route for BMS sold in each consumption country, but does not necessarily reflect the most common route.

### 2.5 Carbon footprint from breastmilk substitute production and consumption

#### 2.5.1 BMS recipe

Based on a review of the literature and of BMS pack labels, the main ingredients of milk-based BMS were identified as skimmed milk, whey protein concentrate, lactose and vegetable oils. A baseline recipe, compliant with the *Codex Alimentarius* standards for protein, fat and carbohydrate content and with at least 50% of protein from whey proteins (Martin et al., 2016), was formulated based on these ingredients (Table 1). Minerals and vitamins were excluded from the assessment, since they constitute only around 2% of dry matter and are unlikely to significantly affect the carbon footprint.

**Table 1:** (Left) Assumed baseline recipe of breastmilk substitute (BMS) and (right) its composition with regard to protein, carbohydrates, fat and ash. The permitted range is shown in brackets.

Ingredients	% of solids	Composition	Unit	Value [*Permitted range*]
Skimmed milk	15	Protein	g/100 kJ	0.60 [0.45–0.70]^a^
Whey protein	10	Whey	% of protein	65% [min 50%]^b^
concentrate		Carbohydrates	g/100 kJ	2.69 [2.30–3.30]^a^
Lactose	50	Fat	g/100 kJ	1.19 [1.05–1.40]^a^
Vegetable oils	25	Ash	g/100 kJ	0.08 [-]^a^
		Energy	kJ/g	21.7

a *Joint FAO/WHO Codex Alimentarius (2007)*.

b Martin et al. (2016).

To assess the uncertainty of the estimated CFPs due to possible variations in recipe formulation, a Monte Carlo simulation (Groen et al., 2014) was performed. For this, BMS recipes based on the main ingredients were randomly generated and for each recipe within the permitted range (Table 1), the CFP was calculated and normalised to an energy content of 21.7 kJ/g, in line with the selected functional unit.

#### 2.5.2 Raw milk

Raw milk is the unprocessed milk leaving dairy farms, which is processed into the milk-based ingredients of BMS. The CFP from raw milk production mainly comprises methane from enteric fermentation in the animal's rumen, but also methane, nitrous oxide and, to a lesser extent, carbon dioxide related to manure management, feed production and energy use (Gerber et al., 2013). There is high variability in the CFP from raw milk production across countries and also across production systems within countries, e.g. between grass-based and intensive systems (Gerber et al., 2013). The raw milk CFP can also differ due to different methodological choices, e.g. where system boundaries are drawn and how allocation is performed. In this study, raw milk was assumed to be produced and further processed into BMS within the same country, i.e. within each of the production case countries. For a consistent CFP of raw milk across the different production case countries, we used CFP values from a study by Hagemann et al. (2011), who based their calculations on typical farms for each country. We compared the CFP values from Hagemann et al. (2011) to those presented in a systematic review by Clune et al. (2017) that included 263 milk CFPs from different production systems and regions and with different embedded methodological choices (Table 2). The CFP values used in this study (from Hagemann et al., 2011) are all within the range of values presented in Clune et al. (2017) except for New Zealand, for which the Hagemann et al. (2011) value is slightly higher. [Table 2 here]

**Table 2:** Raw milk carbon footprint (CFP) values used in this study and values reported in other studies. All CFP values were converted to the common unit of kg CO_2_e per kg energy-corrected milk at farm gate as defined in Hagemann et al. (2011), assuming a raw milk density of 1.035 kg/L.

Source	New Zealand	United States	Brazil	France
Hagemann et al. (2011)^a^	1.09	0.73	1.28	0.99
Clune et al. (2017)^b^	0.77–0.97	0.63–1.86	1.24–1.50	0.77–1.57

a Used in this study.

b Values from Hagemann et al. (2011) excluded.

Allocation of emissions between milk and meat from the dairy production systems was based on the proportion of dairy cow feed intake needed for maintenance and body growth compared with milk production, as described in Cederberg and Stadig (2003), resulting in an allocation factor of 88–94% for milk production in the different case countries (Hagemann et al., 2011).

#### 2.5.3 Vegetable oils

Vegetable oils are the second most important ingredient in BMS. A blend of vegetable oils, including palm, rapeseed, sunflower and soybean oil, is used in BMS production. We had no access to data on the specific blend of oils used in BMS production in each production case country, which also likely varies over time due to market price fluctuations. The oil blend was therefore based on the total national consumption of different vegetable oils in the case countries (Table S4 in Supplementary Data). Data on the CFP of different vegetable oils were taken from Dalgaard et al. (2007) and Schmidt (2015). Allocation of emissions between vegetable oil and residues from oil pressing (often used as animal feed) were based on economic value (Table S5 in Supplementary Data).

#### 2.5.4 Processing

**Fig. 2:**
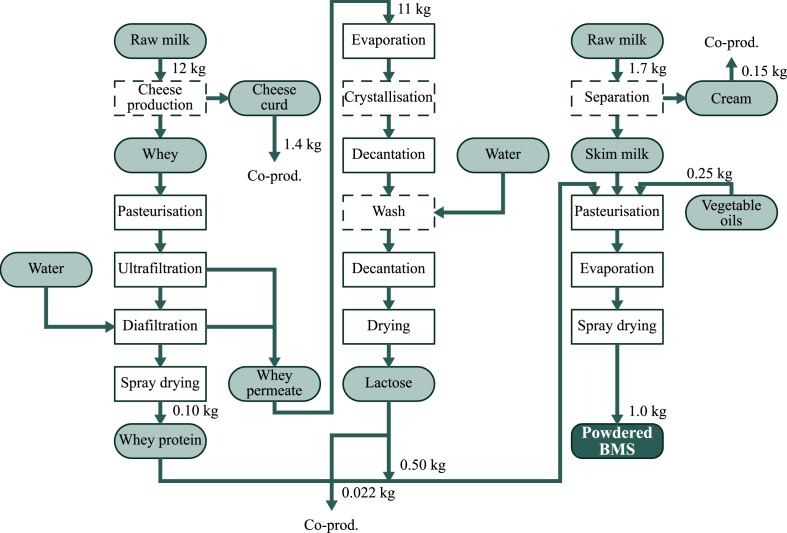
Process steps in the production of powdered breastmilk substitute (BMS). In total, 13.7 kg of raw milk is used for producing 1 kg of BMS, of which 6.6 kg is allocated to the BMS and the rest to other co-products. Numbers represent the wet weight of inputs and co-products in the production of 1 kg of powdered BMS. The inputs in terms of energy used for each processing step is provided in Table S8 in Supplementary Data.

The two main processes used to produce powdered BMS are a dry blending process, where powdered ingredients are mixed into the finished BMS, and a wet mixing and spray drying process, where the dry ingredients (lactose and whey powder) are mixed with liquid ingredients (skimmed milk and oils) and thereafter spray-dried to produce a powder (Guo, 2014). As it is easier to pasteurise liquid ingredients than to ensure that dry ingredients remain free of contaminants, the latter process is preferred (GEA, 2010) and was the method assumed in the present study. Fig. 2 illustrates all relevant processing steps.

The amount of energy needed to produce BMS was estimated by accounting for the energy needed to remove water to produce the dried products, energy for pasteurisation and cooling, and energy for auxiliary equipment not related to the main processes. For electric energy use, we used emission factors for the country-specific electricity mixes (Brander et al., 2011), while for heating energy we assumed the use of natural gas, which is the dominant fuel used in European dairy processing industries (Ramírez et al., 2006). See Tables S5 and S8 in Supplementary Data for more details.

The BMS production process generates a number of co-products (i.e. cheese curd, cream and lactose powder). Processing energy and raw milk therefore need to be allocated between these. As suggested by the International Dairy Federation (IDF, 2015), allocation based on the weight of the dry mass was used here as the default allocation method for the raw milk. Whenever possible, processing energy was allocated to the product being processed. When this was not possible (i.e. when the process was related to two or more co-products), energy use was allocated by the same method as used for the raw milk.

Since the allocation method strongly affected the results, the sensitivity of the results to basing allocation on different properties of the co-products was tested. Thus as well as the default dry mass allocation, we used fat-and-protein allocation as described in Flysjö et al. (2014), allocation based on energy content and allocation based on economic value.

#### 2.5.5 Packaging and transport

Breastmilk substitutes are commonly packaged in tin cans with plastic re-sealable lids, or packs consisting of a plastic bag in a paper box (bag-in-box). No data were available on the relative prevalence of each packaging type in the consumption case countries. To estimate packaging prevalence, we counted the first 100 hits of a Google Image search for “infant formula packaging” and found that 50% of the images depicted either a tin can or a bag-in-box (70% of which were tin cans). We thus assumed that 70% of the BMS was packaged in tin cans and the rest in bag-in-boxes for all consumption case countries. The sensitivity of results to this assumption was tested by also calculating the CFP assuming 100% use of tin cans or bag-in-boxes respectively. Data used for calculating the CFP of packaging are summarised in Table S5 in Supplementary Data.

Processing of BMS and its milk-based ingredients was assumed to occur at three separate locations; a cheese processing plant where whey is a by-product, a processing plant where whey is refined into whey protein and lactose, and a plant where all ingredients are further processed into BMS. Raw milk and whey were assumed to be transported in refrigerated lorries traveling on average 100 km from farm to processing plant and between the cheese processing and BMS processing plants. Dry ingredients (i.e. whey protein and lactose) were assumed to be transported 100 km by non-refrigerated road vehicles. These transport distances are rough estimates in the lack of better data but were deemed feasible.^4^ For the vegetable oils, sea transport and the distance from the main producing country for each oil to each production case country were used (see Table S5 and S6 in Supplementary Data).

#### 2.5.6 Consumption carbon footprint

The consumption CFP included the CFP of BMS production, emissions related to transport from plant to retail outlet, baby bottle manufacture and in-home bottle sterilisation and BMS preparation. Transport was assumed to be mainly by sea from New Zealand to China and from France to Vietnam, while transport from France to UK and transport of BMS produced and consumed in Brazil was assumed to be by road (Table S7 in Supplementary Data).

The number of baby bottles that needed to be sterilised and filled with BMS powder and water was estimated based on the daily energy requirements of infants. It ranges from 504 kJ per kg body weight at birth to 462 kJ per kg body weight at 6 months of age. When adjusted for changes in body weight over the first 6 months of life, infants consume on average 2480 kJ (590 kcal) per day (FAO, 2001) which, divided over six feeds per day, gives 403 kJ or 18.6 g of BMS per serving. Thus, for 1 kg BMS powder, 54 baby bottles need to be sterilised and filled with 0.13 L hot water mixed with BMS powder. Six baby bottles were assumed to be used for 6 months of feeding, which is equivalent to 0.3 bottles per kg BMS powder. Sterilisation was assumed to be undertaken by boiling six bottles at a time in 5 L water (Fig. S4 in Supplementary Data) according to WHO recommendations. To prepare the BMS, water is brought to the boil and mixed with the BMS by hand. The CFP from energy used for sterilisation and preparation was based on average stove types and the energy mix typical for each country (Table S9 in Supplementary Data).

### 2.6 Carbon footprint from breastfeeding

The carbon footprint from breastfeeding (CFP_BF_) equivalent to feeding 1 kg of BMS was estimated as that arising from the production, distribution and cooking of 18 MJ worth of foods representative of the country's average diet (excluding alcoholic beverages). Breastfeeding an infant from birth to 6 months requires mothers to consume on average an additional 500 kcal a day, which equates to 385 MJ over the 6-month period (FAO, 2001). Based on the energy requirements of infants fed BMS (FAO, 2001), a total of 21 kg BMS containing 21.7 kJ/g is required during the same period. Thus, 385/21 = 18 MJ food was assumed to be needed to support a quantity of breastfeeding equivalent to 1 kg of BMS. The CFP associated with this extra food is highly sensitive to the foods that supply the energy required, and ranges from around 0.03 kg CO_2_ equivalents (CO_2_e) per MJ if supplied solely by wheat to 5 kg CO_2_e per MJ if supplied by beef (Clune et al., 2017). Data on the country-specific diets were taken from FAOSTAT food balance sheets^5^ for the years 2011–2013 and the average CFP values arising from the production and distribution of different food items were taken mainly from Clune et al. (2017). See Table S12 in Supplementary Data for a complete list of data and data sources.

To account for emissions from food preparation, we used a standard value of 1.3 MJ heat per kg of food consumed, equivalent to cooking rice (Carlsson-Kanyama and Boström-Carlsson, 2001). We then applied the same country-specific average emissions factors as for the sterilisation and preparation of BMS (Section 2.5.6).

### 2.7 Waste

Data on waste levels throughout the BMS production chain in the different production case countries were taken from Gustavsson et al. (2011) using the “milk” category (Table S10 in Supplementary Data). Data on household waste from food needed and consumed by breastfeeding mothers were also taken from (Gustavsson et al., 2011), subdivided into different food categories. No specific data were available on how much of the prepared BMS is wasted (spilled or discarded) in homes, so BMS wastage was assumed to equal the average household food waste in each consumption case country (Table S10 in Supplementary Data). Due to the uncertainty in this assumption, we evaluated how different BMS waste levels affected the comparison between BMS and breastfeeding CFP.

### 2.8 Land use change

To estimate the land use change (LUC) carbon footprint from BMS consumption and breastfeeding, we used factors derived using a method suggested by Persson et al. (2014). This method allocates emissions from LUC to different products based on their relative contribution to total cropland expansion, rather than only burdening products produced on the actual cleared land an arbitrary number of years after clearing. The justification for this indirect approach is that it is the increased demand for a given commodity *in general* (e.g. soybeans or palm oil) that drives agricultural expansion. Hence all e.g. soybean from a region in which deforestation is taking place should be burdened with the emissions from LUC. Since soybean, palm oil and beef are the internationally traded food commodities most closely associated with LUC, we limited our assessment to these commodities and used LUC-CFP factors from (Henders et al., 2015) (see Table S11 in Supplementary Data).

### 2.9 Sensitivity and uncertainty

#### 2.9.1 One-at-a-time sensitivity analysis

A one-at-a-time sensitivity analysis was performed for both the production CFP (CFP_Prod_) and consumption CFP (CFP_Cons_) to identify model parameters with a large influence on the results. The analysis was performed by varying each parameter by ±10% and observing the change in CFP. For the production phase, the CFP of raw milk and vegetable oil, process energy use (heat and electricity), transport distances and emission factors, the CFP of packaging material and waste levels were varied. For the consumption case, the emissions factors for the different stove fuels, stovetop efficiency, amount of water and associated heating energy used per bottle for sterilisation, amount of BMS prepared per feed and transport distances were varied.

#### 2.9.2 Uncertainty due to variability and uncertainty in model parameters

To estimate the uncertainty range in the final CFP of BMS production and consumption and of breastfeeding, Monte Carlo simulation was used, including uncertainty ranges for a number of selected model parameters identified to substantially influence the resulting CFP. Distributions were assumed based on the literature and our own judgement (Table 3).

**Table 3:** Parameters and estimated uncertainties used in Monte Carlo simulations. CFP = carbon footprint, Prod = production, Cons = consumption, EF = emissions factor, CV = coefficient of variation (= standard deviation/mean x 100).

Parameter	Distribution	Uncertainty	Description
**Production** (CFP_Prod_ and CFP_Cons_)			
Raw milk production EF	Normal	CV: ±20%	Based on variation in data in Clune et al. (2017)
Vegetable oil production EF	Lognormal	CV: ±25%	Based on uncertainty information from Schmidt (2015)
Process energy use, electrical	Triangular	−50% to +80%	Estimated based on benchmarking of Irish dairy powder processing (Geraghty, 2011)
Process energy use, thermal (natural gas)	Triangular	−30% to +60%	
**Consumption** (CFP_Cons_)			
Water per bottle for sterilisation	Triangular	±50%	Our estimate
Stove efficiency	Triangular	±15%	Our estimate
**Breastfeeding** (CFP_BF_)			
Food production and distribution	Lognormal	CV: ±45%^a^	Based on uncertainty information in Clune et al. (2017)
Cooking energy use	Triangular	−30% to +300%	Based on the range between different food products provided in Carlsson-Kanyama and Boström-Carlsson (2001)

a This is the average for all food types, individual values were used for each food type in the calculations (Table S12 in Supplementary Data).

## 3 Results and discussion

### 3.1 Carbon footprint of breastmilk substitute production and consumption

The CFP incurred in producing 1 kg of packaged BMS up to the factory gate (CFP_Prod_) varied between 7.1 and 11 kg CO_2_e per kg BMS, depending on production country (Table 4). The differences between countries are mainly driven by differences in dairy production systems and subsequent variations in the CFP of raw milk production, which constitutes 68–82% of the total CFP_Prod_. For other life cycle stages, there were small variations between countries with the exception of processing emissions for USA, which were notably higher due to its carbon-intensive electricity mix. The Monte Carlo simulation of parameter variability and uncertainty resulted in a coefficient of variation (CV)^6^ of 21–25%.

**Table 4:** Carbon footprint of the production of 1 kg packaged breastmilk substitute (BMS) at the factory gate (CFP_Prod_). The footprint is expressed in kg CO_2_ equivalents (GWP_100_) and uncertainties in the estimates are expressed as ±the standard deviation of the Monte Carlo simulation results.

Production country	Raw milk	Vegetable oils	Processing	Packaging	Transport	Total
New Zealand	7.1 ± 1.4	0.52 ± 0.07	0.72 ± 0.07	0.26	0.57	**9.2 ± 1.4**
United States	4.8 ± 1.0	0.46 ± 0.11	1.0 ± 0.1	0.26	0.55	**7.1 ± 1.0**
Brazil	8.9 ± 1.8	0.54 ± 0.11	0.65 ± 0.06	0.26	0.57	**11 ± 2**
France	6.5 ± 1.3	0.46 ± 0.06	0.62 ± 0.06	0.26	0.56	**8.4 ± 1.3**

The CFP from consumption (i.e. from cradle to bottle) of 1 kg powdered BMS (CFP_Cons_) varied between 11 and 14 kg CO_2_e per kg powdered BMS (Table 5). The consumption phase (i.e. all stages of the life cycle after the factory gate) accounted for some 19–33% of the total CFP_Cons_. Bottle sterilisation was most the important life cycle stage in the consumption phase, contributing 16–27% of total CFP_Cons_. Variations between countries in CFP depended mainly on where the BMS was produced, but the types of stoves and fuels used in each country also influenced CFP_Cons_. In China, 23% of cooking stoves are coal-fired, which led to the largest CFP for sterilisation and preparation among the four consumption countries studied. Emissions related to the production of feeding bottles were negligible and emissions arising from transport from production site to retail were also low. Together, bottle production and transport contributed 1–3% to total CFP_Cons_. The assessment of variability and uncertainty resulted in CV = 12–13%.

**Table 5:** Carbon footprint from consumption of 1 kg powdered breastmilk substitute (BMS) (CFP_Cons_). The footprint is expressed in kg CO_2_ equivalents (GWP_100_) and uncertainties in the estimates are expressed as ± the standard deviation of the Monte Carlo simulation results.

Consumption country (Production country)	BMS prod.	Sterilisation	Preparation	Transport	Bottle prod.	Total
United Kingdom (France)	8.4 ± 1.3	2.4 ± 0.5	0.38 ± 0.02	0.15	0.05	**11 ± 1**
China (New Zealand)	9.2 ± 1.4	3.8 ± 0.8	0.59 ± 0.02	0.20	0.05	**14 ± 2**
Brazil (Brazil)	11 ± 2.0	2.2 ± 0.4	0.33 ± 0.02	0.09	0.05	**14 ± 2**
Vietnam (France)	8.4 ± 1.3	1.8 ± 0.4	0.28 ± 0.01	0.27	0.05	**11 ± 1**

### 3.2 Sensitivity analysis

The one-at-a-time sensitivity analysis performed on the New Zealand production case showed that the raw milk CFP was the parameter with the largest influence on the overall CFP_Prod_, with a 10% increase or decrease leading to an 8% change in CFP_Prod_. For all other parameters tested, the response was below 1% (Fig. S1 in Supplementary Data). The sensitivity analysis for the China consumption case showed that the parameters which most affected the results were (in order of magnitude): stove efficiency, amount of BMS prepared for each feed and amount of water that needed to be heated to sterilise bottles. A 10% increase or decrease in these parameters changed CFP_Cons_ by 2–3% (Fig. S2 in Supplementary Data). New Zealand and China were selected here for illustrative purposes but the other studied case countries showed similar sensitivities but with some regional variation. For example the raw milk CFP influenced results more in the Brazil production case due to a GHG intensive dairy production, and stove efficiency was relatively less important in Brazil and Vietnam due to a cleaner energy mix. Nevertheless stove efficiency was the tested parameter with single largest impact on consumption phase emissions in all studied countries.

Since BMS comprises a mixture of different dairy products (i.e. skimmed milk, whey protein concentrate and lactose powder) with varying economic value and composition with respect to fat, protein, carbohydrates and energy, the allocation method used to divide the CFP of raw milk between these and other dairy processing co-products (i.e. cream and cheese) greatly affected the results. Using the fat and protein content, energy content or economic value of the products as a base for allocation resulted in, on average, a 33, 12 and 36% lower CFP_Cons_, respectively, than the default dry mass allocation method for the case of China. The difference is explained by the importance of lactose, which makes up 50% of BMS and is required to obtain a carbohydrate content similar to breastmilk. Lactose powder is low in fat and protein and relatively cheap compared with other dairy products, which results in low attribution of the raw milk CFP to lactose powder if allocation is based on fat and protein or economic value. If allocation is based on energy or dry mass, a larger share of the raw milk CFP is attributed to lactose powder, leading to a larger CFP from BMS. The allocation method based on fat and protein content proposed by Flysjö et al. (2014) is justified by the fact that these are the constituents of raw milk that usually determine the price farmers receive upon delivery to dairies, and might thus be argued to be the drivers of raw milk production. Such an allocation method results in a very low CFP for lactose, thereby considerably lowering the CFP from BMS. This method is arguably poor in capturing the economic pull exerted by BMS (a high-price product) demand on dairy market dynamics and thus on demand for dairy production.

**Fig. 3:**
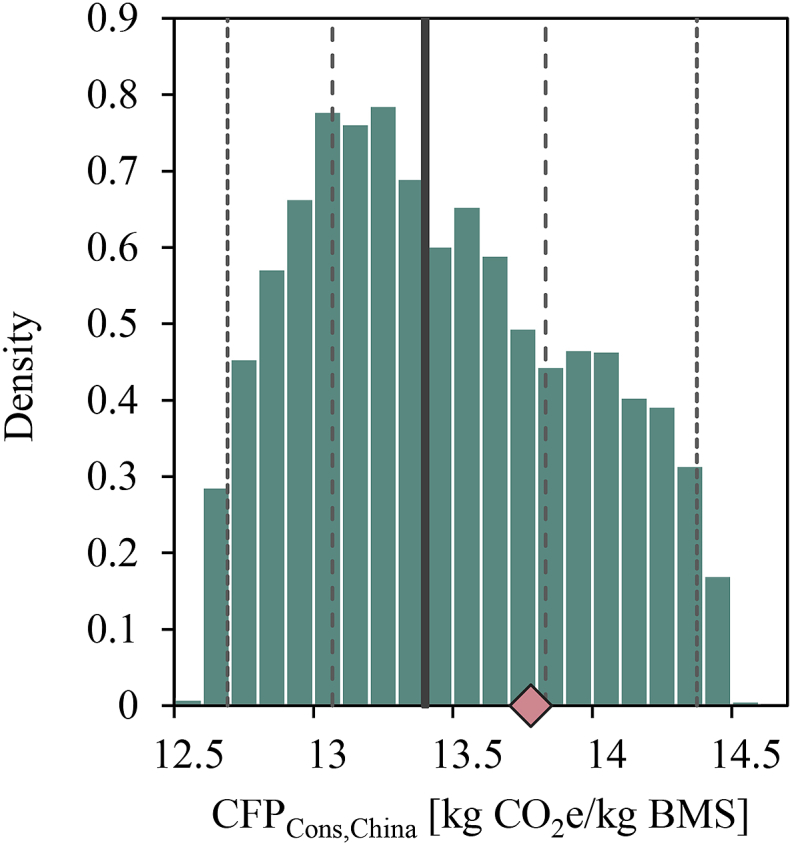
Histogram showing variation in the consumption carbon footprint (CFP_Cons_) for the China case as a result of varying the breastmilk substitute (BMS) recipe. Dashed and dotted lines represent the range including 50% and 95% of simulation outcomes, respectively, and the solid line represents the sample median. The carbon footprint of the baseline recipe is indicated by a red diamond.

Results from simulating different BMS recipes (i.e. different combinations of skimmed milk, whey protein concentrate, lactose and vegetable oils) are shown in Fig. 3 for the China consumption case. The mean CFP_Cons_ of the simulated recipes was 13.5 kg CO_2_e per kg BMS (CV = 3.6%). The average CFP from the simulated recipes was slightly lower than from the assumed baseline recipe. However, the uncertainty in the CFP due to varying the BMS recipe was small compared with the uncertainty arising from variability and uncertainties in model parameters (see Table 5 and Fig. S3 in Supplementary Data). The simulated recipes for which the CFP was smaller than for the baseline recipe contained on average more vegetable oils and fewer dairy-based ingredients.

No data were available for the prevalence of different packaging types used for BMS in the studied countries and we had to resort to a rough estimate for this. Packaging material production did however have a small impact on results and assuming that BMS is packaged solely in bag-in-boxes (low CFP) or tin cans (high CFP) resulted in a 2% reduction or 1% increase in CFP_Prod_ respectively.

Bottle sterilisation had the largest impact on the consumption CFP. Assuming sterilisation in a feeding bottle steam steriliser, instead of the default stove top method, resulted in a 14–23% lower CFP_Cons_, not including emissions related to production of the steam sterilisation equipment. Assuming no sterilisation of feeding bottles (contravening WHO recommendations) resulted in a 16–27% lower CFP_Cons_.

The production of vitamins and minerals used in BMS was omitted from this study and assessments of their contribution to food CFPs are scarce. Jarlbo (2016) has however estimated the CFP from vitamins B_2_, B_12_, D_2_ and potassium carbonate added to oat drinks to be 67, 4.8, 4.8 and 0.3 kg CO_2_e per kg respectively. Adom et al. (2013) states that the CFP from vitamins and amino acids added to cattle feed is 1.1 kg CO_2_e per kg supplement and the Ecoinvent v3.3 database provides a CFP value for ascorbic acid (vitamin C) of 3.1 kg CO_2_e per kg. Using these estimates, and considering that vitamins and minerals constitute around 2% of BMS powder, inclusion of vitamins and minerals would add between 0.006 and 1.3 kg CO_2_e per kg BMS powder (+0.05–12%). Vitamins and minerals could thus contribute non-negligibly to the CFP from BMS, but limited data on the exact composition of minerals and vitamins used in BMS, and on their production processes, made it hard to accurately estimate their contribution to the CFP.

### 3.3 The carbon footprint of feeding BMS compared with breastfeeding

The estimated CFP of the country-specific average diets, including food wasted in the home, ranged between 0.33 and 0.45 kg CO_2_e per MJ of food consumed (Table 6). The additional food recommended for breastfeeding mothers to replace 1 kg of BMS was found to have a CFP between 5.9 and 7.8 kg CO_2_e. The CFP from the UK and Brazil diets was dominated by the quantity of animal-source foods, but for the China and Vietnam diets plant-source foods made up a larger part of the average diet and therefore contributed more to the CFP in these countries. Cooking accounted for between 22 and 34% of the total CFP of the food needed for breastfeeding.

**Table 6:** Carbon footprint from the average diet in each consumption case country per MJ consumed (i.e. after household waste). Uncertainties in the estimates are expressed as ± the standard deviation from the Monte Carlo simulations. BMS = breastmilk substitute.

Case country	Plant-source food	Animal- source food	Cooking	Total	Per kg BMS
United Kingdom	0.074 ± 0.009	0.26 ± 0.05	0.10 ± 0.04	**0.44 ± 0.07**	**6.9 ± 1.0**
China	0.12 ± 0.04	0.15 ± 0.02	0.14 ± 0.05	**0.41 ± 0.07**	**6.5 ± 1.1**
Brazil	0.082 ± 0.011	0.27 ± 0.08	0.10 ± 0.04	**0.45 ± 0.09**	**7.8 ± 1.5**
Vietnam	0.13 ± 0.05	0.13 ± 0.02	0.07 ± 0.03	**0.33 ± 0.06**	**5.9 ± 1.1**

Here, we based our calculations on the recommended additional energy intake for breastfeeding mothers and assumed that these mothers would consume more of the average diets in their country. However, it is not certain that this is actually what breastfeeding mothers do. If it is assumed that the additional energy is provided by lower emitting plant-based foods such as wheat (bread, biscuits etc.) the CFP of breastfeeding will be lower.

**Fig. 4:**
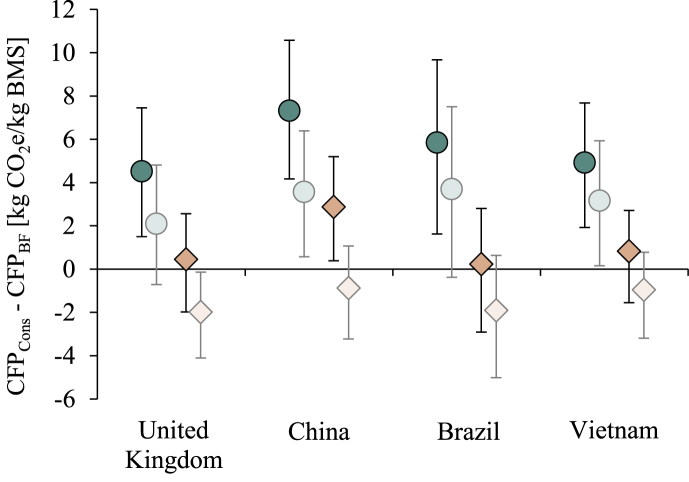
Difference in carbon footprint from breastfeeding (CFP_BF_) and feeding breast milk substitute (BMS) (CFP_Cons_). Values above zero mean that feeding BMS has a higher carbon footprint than breastfeeding, and vice versa. The comparison is presented for the default case (circles), for using fat-and-protein allocation (diamonds), and for excluding bottle sterilisation (shaded circles and diamonds). The whiskers show the 95% confidence intervals.

On average, feeding BMS had a higher climate impact than breastfeeding in all countries studied, when using the default allocation and sterilisation methods (Fig. 4). After accounting for uncertainty in CFP, CFP_Cons_ was still higher than CFP_BF_ in more than 99% of the cases for all case countries. Using the alternative fat-and-protein allocation method resulted in emissions from feeding BMS exceeding those from breastfeeding in 66, 97, 59 and 76% of the Monte Carlo runs in UK, China, Brazil and Vietnam, respectively. Feeding a baby BMS for 6 months requires 21 kg of BMS, generating a climate impact of 226–288 kg CO_2_e in total, while the range for breastfeeding is about 123–162 kg CO_2_e, resulting in a net benefit of breastfeeding of 95–153 kg CO_2_e compared with exclusively feeding BMS for the first 6 months.

**Fig. 5:**
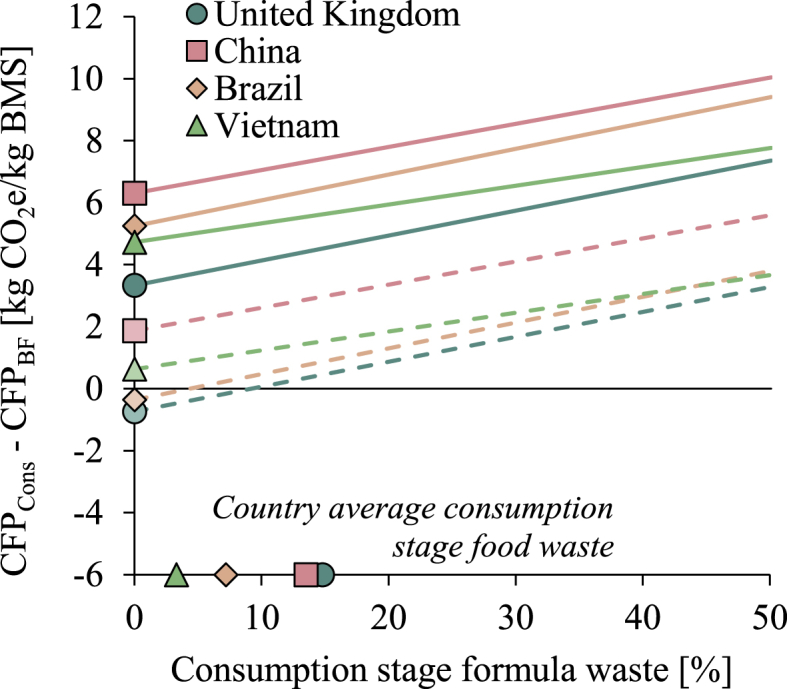
Ratio between the carbon footprint of breastfeeding (CFP_BF_) and feeding breast milk substitute (BMS) (CFP_Cons_) under different assumptions on amount of BMS wasted at the consumer stage. Solid lines represent the comparison using the default dry mass allocation method and dashed lines the alternative fat-and-protein allocation method. Values above zero mean that feeding BMS has a higher carbon footprint than breastfeeding, and vice versa. The cases of United Kingdom, China, Brazil and Vietnam are represented by blue circles, red squares, yellow diamonds and green triangles, respectively. The symbols at the bottom of the diagram indicate the country-average, consumption-stage food waste used to estimate BMS waste levels in the default calculations.

Fig. 5 shows how varying assumptions about food wastage associated with feeding BMS affected the comparison between breastfeeding and feeding BMS. Since CFP_BF_ was defined as emissions arising from providing sufficient breastmilk to match 1 kg powdered BMS ready for consumption, the level of BMS wasted at the consumption stage affected the comparison. When the fraction of BMS wasted increased (i.e. less of the prepared BMS is actually consumed), the amount of breastmilk needed to replace each kg of BMS powder (some of which is not consumed) decreased and thus also CFP_BF_. The analysis showed that breastfeeding carried a lower CFP for all BMS waste scenarios and for both allocation methods except for the fat-and-protein allocation method when waste levels are below 5% and 10% in Brazil and UK, respectively. In China and Vietnam, the average carbon footprint of feeding BMS exceeded that of breastfeeding, irrespective of allocation method and assumed BMS wastage.

Changing the characterisation factor for methane from 25 (IPCC AR4) to 28 (IPCC AR5) kg CO_2_e per kg CH_4_ resulted in a 4–5% increase in CFP_Cons_ and a 2–4% increase in CFP_BF_ (due to the meat and dairy content of the mothers’ diets). Including carbon feedback loops (i.e. using 34 kg CO_2_e per kg CH_4_) resulted in an increase of 11–15% and 6–12% for CFP_Cons_ and CFP_BF_ respectively. Since CFP_Cons_ and CFP_BF_ had similar sensitivity to the methane characterisation factor, the comparison between breastfeeding and feeding BMS was robust to changes in this characterisation factor.

The assumed transport distances between dairy farms and processing plants were based on rough estimates due to lack of better data. The relationship between CFP_Cons_ and CFP_BF_ was however found to be robust to changes in transport distance for all cases under the default allocation method.

The contribution of LUC to the carbon footprint of BMS was small, adding between 0.5 and 0.7% to CFP_Cons_. For CFP_BF_, the results were highly variable. Inclusion of LUC doubled the CFP of the Brazil diet, due to considerable consumption of local beef associated with forest clearing for pasture. For the other case countries, inclusion of LUC added no more than 2% to the total CFP.

## 4 Conclusions

The results obtained in this study indicate that breastfeeding has a consistently lower carbon footprint than using BMS. This was true for all four countries studied in the assessment: UK, China, Brazil and Vietnam, where the impact of breastfeeding was 40%, 53%, 43% and 46% lower, respectively, than that arising from using BMS. However, the results were sensitive to allocation of emissions between different dairy co-products and using alternative allocation methods resulted in a 12%–36% smaller CFP from BMS. If allocation was based on the fat and protein content of dairy co-products (i.e. allocating few emissions to lactose) and no sterilization of feeding bottles was assumed (in contradistinction to WHO recommendations) the CFP from BMS was smaller than that of breastfeeding in the studied case countries. This study only assessed the climate impact of different infant feeding approaches, but food production also affects the environment in many other ways, including via pollution and contamination of waterways and soils, biodiversity loss and use of limited or non-renewable resources such as land, water and fossil fuels. Ideally, a full comparison of the environmental impact of BMS versus breastfeeding would also take these into account, but this is a difficult task, made harder by the fact that many of the impacts that arise are localised, whereas the climate impacts assessed here are global. In this study BMS was compared to breastfeeding assuming that the two are functionally equivalent and substitutable. However, this is often not the case. On the one hand a large (although recently partly contested) evidence base points towards positive health and developmental outcomes of breastfeeding as compared to using BMS. But, on the other hand breastmilk substitutes are a necessity in cases when, for medical or other reasons, breastfeeding is not a possibility. Care should therefore be taken when comparing the climate impact of these two “products” with partly diverging functions.

## Declarations of interest

None.

## Disclaimer

The authors alone are responsible for the views expressed in this article and they do not necessarily represent the decisions, policy or views of the institutions with which they are affiliated.

## Funding

This work was supported by the World Health Organization [grant number 201737339] and the Department of Energy and Technology, Swedish University of Agricultural Sciences.

## Footnotes

1 http://www.who.int/maternal_child_adolescent/topics/child/nutrition/breastfeeding/en, accessed 17 May 2018.

2 FAOSTAT Database, http://www.fao.org/faostat/en/#data, accessed 25 September 2018.

3 UN Comtrade Database, https://comtrade.un.org/data/, accessed 13 March 2018.

4 Anna Flysö, Life Cycle Sustainability Manager, Arla Foods, Denmark (personal communication, February 5, 2019).

5 FAOSTAT database http://www.fao.org/faostat/en/#data, accessed 13 February 2017.

6 Defined as the standard deviation of the Monte Carlo simulation results divided by the mean (σ/x̄∙100).
