# Supplementary material for: The carbon footprint of breastmilk substitutes in comparison with breastfeeding
Source: J Clean Prod. 2019 Jun 10;222:436–45. doi: 10.1016/j.jclepro.2019.03.043 (PMC6472111; doi:10.1016/j.jclepro.2019.03.043)
Supplement: Multimedia Component 1 [file mmc1.docx]

**Supplementary Data – The carbon footprint of breastmilk substitutes in comparison with breastfeeding**

Table S1. Inventory for BMS production per 1 kg BMS powder

|  | Unit | New Zealand | United States | Brazil^1^ | France |
| --- | --- | --- | --- | --- | --- |
| Raw milk | kg ECM | 2.61 | 2.61 | 2.79 | 2.61 |
| Soybean oil | kg | 0.114 | 0.232 | 0.221 | 0.0725 |
| Rapeseed oil | kg | 0.082 | 0.019 | 0.000 | 0.057 |
| Sunflower seed oil | kg | 0.033 | 0.003 | 0.006 | 0.114 |
| Palm oil | kg | 0.025 | 0.000 | 0.029 | 0.009 |
| Electricity | kWh | 0.685 | 0.685 | 0.690 | 0.685 |
| Natural gas | MJ | 7.048 | 7.048 | 7.106 | 7.048 |
| Canning steel | kg | 0.068 | | | |
| LDPE pouch | kg | 0.027 | | | |
| Box board | kg | 0.030 | | | |
| Sea freight | tonne∙km | 2.75 | 0.08 | 0.46 | 1.56 |
| Road freight | tonne∙km | 0.16 | 0.21 | 0.21 | 0.16 |
| Refrigerated road freight milk | tonne∙km | 0.75 | 0.75 | 0.78 | 0.75 |

^1^More ingredients are needed in Brazil, as waste levels are slightly higher than in the other countries.

Table S2. Inventory for BMS consumption per 1 kg BMS powder

|  | Unit | United Kingdom (FR) | China (NZ) | Brazil (BZ) | Vietnam (FR) |
| --- | --- | --- | --- | --- | --- |
| BMS powder | kg | 1.0 | | | |
| Packaging | kg | 0.125 | | | |
| Feeding bottles (HDPE) | kg | 0.027 | | | |
| Electricity | kWh | 1.897 | 0.559 | 0.025 | 0.020 |
| Natural gas | MJ | 17.83 | 9.07 | 25.09 | 9.26 |
| Kerosene | MJ | 0.00 | 0.10 | 0.00 | 0.42 |
| LPG | MJ | 0.00 | 0.00 | 0.00 | 0.00 |
| Hard coal | MJ | 0.00 | 16.96 | 1.04 | 3.01 |
| Charcoal | MJ | 0.00 | 0.00 | 0.23 | 2.02 |
| Wood | MJ | 0.00 | 26.32 | 9.61 | 54.96 |
| Sea freight | tonne∙km | 0.00 | 11.70 | 0.00 | 17.77 |
| Road freight | tonne∙km | 1.14 | 0.46 | 0.69 | 0.46 |

*Table S3. Composition of dairy and non-dairy ingredients and inputs and co-products from production of powdered dairy ingredients. These figures were used for allocation and calculation of BMS protein, lactose and fat content*

|  | Protein |  | Lactose | Fat | Ash | Price |
| --- | --- | --- | --- | --- | --- | --- |
|  | g/100 g | %whey | g/100 g | g/100 g | g/100 g | €/ton |
| *Dairy ingredients used in infant formula:* | | | |  |  |  |
| Skim milk | 36 (1) | 14.5 | 51 (1) | 8.2 (1) | 0.8 (1) | 1700 (4) |
| WPC80 | 77 (1) | 100 | 9.0 (1) | 6.0 (1) | 4.0 (1) | 1700 (4) |
| Lactose (refined) | 0.0 (1) | 100 | 99.5 (1) | 0.0 (1) | 0.2 (1) | 600 (4) |
| *Inputs and co-products:* | | | |  |  |  |
| Raw milk | 3.5 (1) |  | 4.5 (1) | 3.5 (1) | 0.7 (1) |  |
| Cream | 2.0 (2) |  | 2.8 (2) | 37 (2) | 0.5 (2) | 1900 (4) |
| Whey permeate | 0.1 (1) |  | 4.9 (1) | 0.0 (1) | 0.5 (1) | 33.1 (4)* |
| Cheese curd | 25 (3) |  | 1.6 (3) | 27 (3) | 2.4 (3) | 2800 (4)** |
| *Non-dairy ingredients used in infant formula:* | | | |  |  |  |
| Vegetable oil | 0.0 |  | 0.0 | 100 | 0.0 |  |

(1) Smith (2008).
(2) milkINGREDIENTS.ca, <http://www.milkingredients.ca/index-eng.php?link=114>, accessed 25 Sept 2018.
(3) Hui (2006).
(4) Average prices for 2015-2017 according to <http://www.clal.it>, accessed 25 Sept 2018.
*Calculated based on lactose, which is the saleable product of whey permeate (0.049 kg lactose per kg whey permeate).
**Calculated based on cheddar cheese (0.88 kg cheddar cheese per kg cheese curd).

Table S4. Contribution by the four main oils to total vegetable oil consumption (%) in each production case country. Figures refer to the years 2009-2013. The main producing and exporting countries are listed from highest to lowest based on 2013 production and export quantities. Source: FAOSTAT Database, <http://www.fao.org/faostat/en/#data>, accessed 08 Dec 2017

|  | New Zealand | United states | Brazil | France | Main producing countries | Main exporting countries |
| --- | --- | --- | --- | --- | --- | --- |
| Soybean oil | 35 | 77 | 77 | 19 | China, United States, Brazil | Argentina, Brazil, United States |
| Rape and mustard oil | 25 | 6.2 | 0 | 15 | China, Germany, Canada | Canada ,Germany, Belgium |
| Sunflower seed oil | 10 | 0.9 | 2.0 | 30 | Ukraine, Russia, Argentina | Ukraine, Russia, Netherlands |
| Palm and palm kernel oil | 8.1 | 0.2 | 10 | 4.5 | Indonesia, Malaysia, Thailand | Indonesia, Malaysia, Netherlands |
|  |  |  |  |  |  |  |
| Total | 78 | 85 | 89 | 69 |  |  |

Table S5. Emissions factors used in the study, expressed as kg CO_2_e according to IPCC 2007 GWP_100_

| Emissions factor | Geography | Unit | Value | Source/Description |
| --- | --- | --- | --- | --- |
| *Agricultural production*: | |  |  |  |
| Raw milk | New Zealand | /kg ECM | 1.09 | Hagemann et al. (2011). Allocation based on energy expenditure. |
|  | United States | /kg ECM | 0.73 | “ |
|  | Brazil | /kg ECM | 1.28 | “ |
|  | France | /kg ECM | 0.99 | “ |
| Vegetable oils |  |  |  |  |
| Soybean oil | Argentina | /kg oil | 1.82 | Dalgaard et al. (2007). Economic allocation AF=0.31 |
| Rapeseed oil | EU27 | /kg oil | 1.91 | Schmidt (2015). Adjusted to economic allocation AF=0.73 |
| Sunflower oil | Ukraine | /kg oil | 1.60 | Schmidt (2015). Adjusted to economic allocation AF=0.73 (assumed equal to rapeseed oil) |
| Palm oil | Indonesia/ Malaysia | /kg oil | 4.27 | Schmidt (2015). Adjusted to economic allocation AF=0.98 |
|  |  |  |  |  |
| *Processing:* |  |  |  |  |
| Grid electricity | New Zealand | /kWh | 0.215 | Brander et al. (2011) |
|  | United States | /kWh | 0.589 | “ |
|  | Brazil | /kWh | 0.110 | “ |
|  | France | /kWh | 0.0759 | “ |
| Natural gas | Global | /MJ | 0.072 | Prod and distribution: Ecoinvent v3.3, Combustion: (IPCC, 2006) |
|  |  |  |  |  |
| *Transport:* |  |  |  |  |
| Sea freight | Global | /tonne∙km | 0.0115 | Ecoinvent v3.3 |
| Road freight | Global | /tonne∙km | 0.132 | “ |
| Road freight, refrigerated | Global | /tonne∙km | 0.456 | “ |
|  |  |  |  |  |
| *Packaging:* |  |  |  |  |
| Canning steel | Global | /kg packaging | 1.60 | Wallman and Nilsson (2011). 69% recycled |
| Boxboard with print | Global | /kg packaging | 0.594 | Ecoinvent v3.3 |
| LDPE packaging film | Global | /kg packaging | 2.90 | “ |
|  |  |  |  |  |
| *Stove-top fuels:* |  |  |  |  |
| Grid electricity | United Kingdom | /kWh | 0.550 | Brander et al. (2011) |
|  | China | /kWh | 1.04 | “ |
|  | Brazil | /kWh | 0.110 | “ |
|  | Viet Nam | /kWh | 0.473 | “ |
| Natural gas | Global | /MJ | 0.072 | Prod and distribution: Ecoinvent v3.3, Combustion: (IPCC, 2006) |
| Kerosene | Global | /MJ | 0.085 | “ |
| LPG | Global | /MJ | 0.078 | “ |
| Coal | Global | /MJ | 0.125 | “ |
| Charcoal | Global | /MJ | 0.055 | “ |
| Wood | Global | /MJ | 0.009 | “ (no emissions assumed from production and distribution) |

Table S6. Assumed transport distances from vegetable oil production plant to BMS production plant. Sea distances calculated with the [www.ecotransit.org](http://www.ecotransit.org) online tool

|  | New Zealand | | United States | | Brazil | | France | |
| --- | --- | --- | --- | --- | --- | --- | --- | --- |
| Soybean oil | Argentina | | (domestic) | | (domestic) | | Brazil | |
|  | Sea | Road | Sea | Road | Sea | Road | Sea | Road |
|  | 10.9k | 400 | NA | 600 | NA | 600 | 9.3k | 400 |
| Rape and mustard oil | Canada | | Canada | | Canada | | Germany | |
|  | Sea | Road | Sea | Road | Sea | Road | Sea | Road |
|  | 11.5k | 400 | 2.2k | 400 | 16.1k | 400 | 1.2k | 400 |
| Sunflower seed oil | Argentina | | Argentina | | Argentina | | Ukraine | |
|  | Sea | Road | Sea | Road | Sea | Road | Sea | Road |
|  | 10.9k | 400 | 13.4k | 400 | 1.7k | 400 | 6.0k | 400 |
| Palm and palm kernel oil | Indonesia | | Indonesia | | Indonesia | | Indonesia | |
|  | Sea | Road | Sea | Road | Sea | Road | Sea | Road |
|  | 8.6k | 400 | 14.7k | 400 | 15.7k | 400 | 15.2k | 400 |

Table S7. Assumed transport distances from BMS production plant to retail. Sea distances calculated with the [www.ecotransit.org](http://www.ecotransit.org) online tool

|  | United Kingdom | | China | | Brazil | | Vietnam | |
| --- | --- | --- | --- | --- | --- | --- | --- | --- |
| BMS powder | France | | New Zealand | | Brazil | | France | |
|  | Sea | Road | Sea | Road | Sea | Road | Sea | Road |
|  | NA | 1000 | 10.4k | 400 | NA | 600 | 15.8k | 400 |

Table S8. Electrical and thermal energy use for different process steps

|  | Grid electricity | Natural gas | Source |
| --- | --- | --- | --- |
|  | [kWh] | [MJ] |  |
| Per kg water removed: |  |  |  |
| Evaporation | 0.0069 | 0.40 | GEA (2010) |
| Filtration | 0.0069 | - | Ramírez et al. (2006) |
| Spray + fluid bed drying | 0.10 | 7.0 | GEA (2010) |
| Fluid bed drying | 0.014 | 0.29 | GEA (2010) |
| Decanter centrifuge | 0.0024 | - | Rak (2011) |
| Per kg product: |  |  |  |
| Pasteurisation + cooling | 0.025 | - | Kažimírová (2013) |
| Utilities^1^ | 0.186 | - | Gearghty (2011) |

^1^Pumps for water and cooling systems etc.

Table S9. End use efficiency of different stove-top fuels and their prevalence in the consumption case countries

|  | End use efficiency (1) | Prevalence | | | |
| --- | --- | --- | --- | --- | --- |
|  |  | United Kingdom (2) | China (3) | Brazil (3) | Vietnam (3) |
| Electric | 0.80 | 0.38 | 0.11 | 0.005 | 0.004 |
| Natural gas | 0.50 | 0.62 | 0.31 | 0.87 | 0.32 |
| Kerosene | 0.45 | - | 0.003 | - | 0.013 |
| LPG | 0.50 | - | - | - | - |
| Coal | 0.25 | - | 0.29 | 0.018 | 0.052 |
| Charcoal | 0.25 | - | - | 0.004 | 0.035 |
| Wood | 0.15 | - | 0.27 | 0.10 | 0.57 |

(1) Hager and Morawicki (2013).
(2) UK Department of Energy & Climate Change (2013).
(3) Global alliance for Clean Cookstoves, Country-level Data, <http://cleancookstoves.org/country-profiles/>, accessed 25 Sept 2018.

Table S10. Waste factors used for the different BMS production stages and in the consumption stage in the different regions (Gustavsson et al., 2011)

| Food chain stage | Country |  |  |  |
| --- | --- | --- | --- | --- |
| *Production:* | New Zealand | United States | Brazil | France |
| Agricultural production | - | - | - | - |
| Postharvest handling and storage | 0.5% | 0.5% | 6% | 0.5% |
| Processing and packaging | 1.2% | 1.2% | 2% | 1.2% |
| Distribution | - | - | - | - |
|  |  |  |  |  |
| *Consumption*: | United Kingdom | China | Brazil | Vietnam |
| Consumption | 15% | 14% | 7.2% | 3.3% |

Table S11. Data used for estimating CO_2_ emissions from land use change (LUC) due to use of soy in animal production (both for milk used in infant formula and animal products in the breastfeeding mothers assumed diets), imported South American beef

|  | United Kingdom | China | Brazil | Vietnam | Description |
| --- | --- | --- | --- | --- | --- |
| *Beef:* |  |  |  |  |  |
| Import | 33% | 7.6% | 0.5% | 42% | Calculated from (1) using imp/(imp+prod) |
| LUC of import | 5% | 34% | 100% | 0.3% | Based on imports from South America according to (2) |
| LUC of domestic | 0% | 0% | 100% | 0% | Our estimate |
|  |  |  |  |  |  |
| *Soy in animal feed (kg LUC soy used per kg product):* | | | | | |
| Beef | 0.2 | 0.2 | 0.2 | 0 | Based on soy used in |
| Pork | 0.3 | 0.3 | 0.3 | 0 | animal farming in the |
| Chicken | 0.6 | 0.6 | 0.6 | 0 | Netherlands (3) |
| Eggs | 0.3 | 0.3 | 0.3 | 0 |  |
| Milk | 0.02 | 0.02 | 0.02 | 0 |  |
|  |  |  |  |  |  |
| *LUC emissions factor:* | Beef | Soy | Palm oil |  |  |
| (kg CO2e/kg) | 81.3 | 0.7 | 2.1 |  | From (4), assuming Brazilian beef and soy and Indonesian palm oil. |

(1) FAOSTAT Database, Food balance sheets, <http://www.fao.org/faostat/en/#data>, accessed 13 Feb 2017.
(2) UN Comtrade Database, <https://comtrade.un.org/data/>, accessed 25 Sept 2018.
(3) Soya consumption in the Netherlands, <http://edepot.wur.nl/157676>, accessed 26 Sept 2018.
(4) Henders et al. (2015).

Table S12. Data used for estimating food consumption CFP. Consumption is before losses at the consumer stage. Meat is bone-free meat

| Food item | Food consumption (kg/capita/year) | | | | Emissions factor* (kg CO_2_e/kg) | |
| --- | --- | --- | --- | --- | --- | --- |
|  | United Kingdom | China | Brazil | Vietnam | Mean | SD |
| *Animal fats:* |  |  |  |  |  |  |
| Butter, ghee | 3.3 | 0.12 | 0.48 | 0.17 | 9.25 | 7.37 |
| Cream | 0.31 | 0.013 | - | 0.010 | 5.64 | 1.62 |
| Fats, animals, raw | 2.7 | 2.0 | 3.0 | 2.2 | 9.25 | 7.37 |
| Fish, body oil | - | - | - | - | 3.49 | 3.62 |
| Fish, liver oil | - | - | - | - | 3.49 | 3.62 |
| *Aquatic products, other:* |  |  |  |  |  |  |
| Aquatic animals, others | 0.010 | 0.68 | 0.0067 | 0.33 | 3.49 | 3.62 |
| Aquatic plants | - | 9.02 | - | - |  |  |
| Meat, aquatic mammals | - | - | - | - | 3.49 | 3.62 |
| *Cereals - excluding beer:* |  |  |  |  |  |  |
| Barley and products | 0.74 | 0.17 | - | - | 0.5 | 0.22 |
| Cereals, other | 0.30 | 0.28 | 0.78 | 0.053 | 0.5 | 0.22 |
| Maize and products | 2.9 | 7.0 | 27 | 10 | 0.5 | 0.22 |
| Millet and products | - | 0.56 | - | - | 0.5 | 0.22 |
| Oats | 6.2 | 0.13 | 1.9 | - | 0.5 | 0.22 |
| Rice (milled equivalent) | 6.8 | 78 | 33 | 144 | 2.55 | 1.29 |
| Rye and products | 0.37 | 0.093 | 0.027 | - | 0.5 | 0.22 |
| Sorghum and products | - | 1.0 | - | - | 0.5 | 0.22 |
| Wheat and products | 98 | 63 | 53 | 11 | 0.5 | 0.22 |
| *Eggs:* |  |  |  |  |  |  |
| Eggs | 11 | 19 | 8.9 | 3.7 | 3.46 | 1.21 |
| *Fish, seafood:* |  |  |  |  |  |  |
| Cephalopods | 0.17 | 0.82 | 0.04 | 1.5 | 7.13 | 2.4 |
| Crustaceans | 3.8 | 3.8 | 0.63 | 2.9 | 7.8 | 12.37 |
| Demersal fish | 5.4 | 2.1 | 2.2 | 0.044 | 3.49 | 3.62 |
| Freshwater fish | 2.0 | 9.9 | 3.0 | 8.2 | 3.49 | 3.62 |
| Marine fish, other | 0.059 | 0.89 | 0.39 | 8.0 | 3.49 | 3.62 |
| Molluscs, other | 0.71 | 5.4 | 0.12 | 1.2 | 3.49 | 3.62 |
| Pelagic fish | 2.2 | 0.25 | 0.66 | 0.62 | 3.49 | 3.62 |
| *Fruits - excluding wine:* |  |  |  |  |  |  |
| Apples and products | 27 | 20 | 5.2 | 0.93 | 0.42 | 0.32 |
| Bananas | 16 | 7.0 | 30 | 18 | 0.42 | 0.32 |
| Citrus, other | 0.30 | 3.1 | - | - | 0.42 | 0.32 |
| Dates | 0.23 | 0.11 | - | - | 0.42 | 0.32 |
| Fruits, other | 24 | 35 | 24 | 34 | 0.42 | 0.32 |
| Grapefruit and products | 3.8 | 2.1 | 0.33 | 4.6 | 0.42 | 0.32 |
| Grapes and products (excl. wine) | 11 | 5.7 | 4.8 | 0.64 | 0.42 | 0.32 |
| Lemons, limes and products | 2.1 | 1.2 | 4.4 | - | 0.42 | 0.32 |
| Oranges, mandarins | 38 | 15 | 35 | 5.5 | 0.42 | 0.32 |
| Pineapples and products | 3.5 | 1.1 | 9.8 | 5.3 | 0.42 | 0.32 |
| Plantains | 0.41 | - | - | - | 0.42 | 0.32 |
| *Meat:* |  |  |  |  |  |  |
| Bovine meat | 13 | 3.5 | 27 | 5.2 | 26.61 | 12.47 |
| Meat, other | 1.1 | 0.80 | 0.075 | 0.20 | 15.4 | 6.94 |
| Mutton & goat meat | 2.9 | 2.0 | 0.40 | 0.090 | 25.58 | 11.93 |
| Pig meat | 16 | 22 | 7.4 | 20 | 5.77 | 1.63 |
| Poultry meat | 24 | 10 | 32 | 10 | 3.65 | 1.72 |
| *Milk - excluding butter:* |  |  |  |  |  |  |
| Milk - excluding butter | 236 | 33 | 150 | 16 | 1.29 | 0.58 |
| *Miscellaneous:* |  |  |  |  |  |  |
| Infant food | 1.2 | 0.073 | - | 0.12 |  |  |
| Miscellaneous | - | - | - | - |  |  |
| *Offal:* |  |  |  |  |  |  |
| Offal, edible | 2.0 | 3.5 | 2.3 | 4.2 | 16.19 | 7.05 |
| *Oilcrops:* |  |  |  |  |  |  |
| Coconuts - incl. copra | 1.1 | 0.33 | 13.3 | 3.5 | 0.49 | 0.04 |
| Cottonseed | - | - | - | - | 0.49 | 0.04 |
| Groundnuts (shelled eq) | 1.7 | 3.4 | 0.34 | 2.7 | 0.99 | 0.48 |
| Oilcrops, Other | 0.017 | - | 0.0067 | - | 0.49 | 0.04 |
| Olives (including preserved) | 0.65 | - | 0.51 | - | 0.63 | 0.22 |
| Palm kernels | - | - | - | - | 0.49 | 0.04 |
| Rape and mustardseed | - | - | - | - | 0.49 | 0.04 |
| Sesame seed | 0.10 | 0.32 | 0.047 | - | 0.88 |  |
| Soyabeans | 0.073 | 3.9 | 3.7 | 5.2 | 0.49 | 0.04 |
| Sunflower seed | - | - | 0.040 | - | 0.49 | 0.04 |
| *Pulses:* |  |  |  |  |  |  |
| Beans | 0.42 | 0.080 | 16 | 1.8 | 0.51 | 0.45 |
| Peas | 1.6 | 0.59 | 0.23 | - | 0.51 | 0.45 |
| Pulses, other and products | 1.0 | 0.69 | 0.14 | 1.3 | 0.51 | 0.45 |
| *Spices:* |  |  |  |  |  |  |
| Cloves | - | - | - | 0.030 |  |  |
| Pepper | 0.17 | 0.023 | 0.053 | 0.34 | 0.66 | 0.27 |
| Pimento | 0.14 | 0.14 | - | 0.96 | 0.66 | 0.27 |
| Spices, other | 0.75 | 0.15 | 0.060 | 0.54 |  |  |
| *Starchy roots:* |  |  |  |  |  |  |
| Cassava and products | - | 1.9 | 40 | 8.2 | 0.18 | 0.08 |
| Potatoes and products | 102 | 41 | 18 | 4.2 | 0.18 | 0.08 |
| Roots, other | - | 1.1 | - | - | 0.2 | 0.15 |
| Sweet potatoes | - | 24 | 1.0 | 5.1 | 0.18 | 0.08 |
| Yams | 0.11 | - | 0.58 | - | 0.18 | 0.08 |
| *Stimulants:* |  |  |  |  |  |  |
| Cocoa beans and products | 3.4 | 0.073 | 1.1 | 0.10 | 0.43 | 0.45 |
| Coffee and products (1) | 2.6 | 0.073 | 3.3 | 0.010 | 3 |  |
| Tea (including mate) (1) | 2.0 | 1.0 | 2.2 | 0.79 | 3 |  |
| *Sugar & sweeteners (2):* |  |  |  |  |  |  |
| Honey | 0.63 | 0.25 | 0.093 | - |  |  |
| Sugar (raw equivalent) | 38 | 6.7 | 40 | 9.6 | 0.87 |  |
| Sugar non-centrifugal | - | 0.31 | 2.4 | - | 0.87 |  |
| Sweeteners, other | 1.3 | 0.12 | 0.53 | 0.55 | 0.87 |  |
| *Sugar crops:* |  |  |  |  |  |  |
| Sugar beet | - | - | - | - | 0.29 |  |
| Sugar (raw equivalent) (2) | - | 0.023 | 16 | 12 | 0.87 |  |
| *Treenuts:* |  |  |  |  |  |  |
| Nuts and products | 2.2 | 2.7 | 0.80 | 4.3 | 1.2 | 0.93 |
| *Vegetable oils (3):* |  |  |  |  |  |  |
| Coconut oil | 0.083 | 0.13 | 0.013 | 1.1 | 4.53 |  |
| Cottonseed oil | - | 0.55 | 1.3 | - | 4.53 |  |
| Groundnut oil | 0.037 | 0.66 | 0.010 | 0.33 | 4.53 |  |
| Maize germ oil | 0.42 | 0.17 | 0.28 | - | 4.53 |  |
| Oilcrops oil, other | 0.52 | 0.083 | - | 0.28 | 4.53 |  |
| Olive oil | 0.96 | 0.033 | 0.36 | 0.010 | 4.53 |  |
| Palm oil | 0.56 | 2.0 | 1.8 | - | 4.53 |  |
| Palm kernel oil | 0.14 | 0.060 | - | - | 4.53 |  |
| Rape and mustard oil | 8.3 | 1.6 | - | 0.033 | 4.53 |  |
| Ricebran oil | - | 0.073 | 0.080 | 0.12 | 4.53 |  |
| Sesame seed oil | 0.037 | 0.17 | 0 | 0.070 | 4.53 |  |
| Soyabean oil | 4.2 | 2.1 | 14 | 0.99 | 4.53 |  |
| Sunflower seed oil | 2.3 | 0.15 | 0.37 | 0.010 | 4.53 |  |
| *Vegetables:* |  |  |  |  |  |  |
| Onions | 10 | 14 | 6.8 | 4.9 | 0.37 | 0.39 |
| Tomatoes and products | 22 | 30 | 19 | - | 0.37 | 0.39 |
| Vegetables, other | 64 | 298 | 26 | 117 | 0.37 | 0.39 |

*Emissions factors from Clune et al. (2017) except for:
(1) Röös (2014).
(2) Ecoinvent v3.3 Average of global market for sugar from sugar beet and sugar cane.
(3) Ecoinvent v3.3 Global market for vegetable oil.


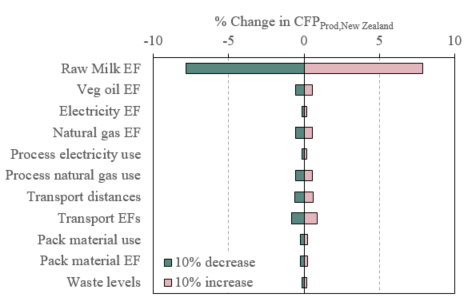


Figure S1. Sensitivity of the production CFP for the New Zealand case to changes in selected parameters. Light (pink) bars represent a 10% increase in the parameter value and dark (green) bars represent a 10% decrease.


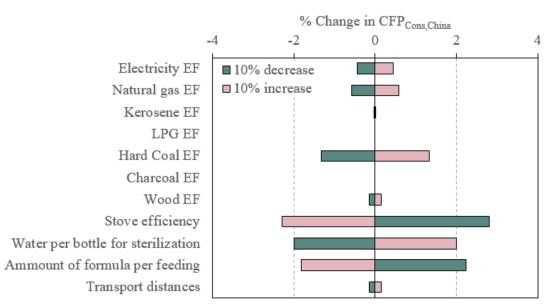


Figure S2. Sensitivity of the consumption CFP for the Chinese case to changes in selected parameters. Light (pink) bars represent a 10% increase in the parameter value and dark (green) bars represent a 10% decrease.


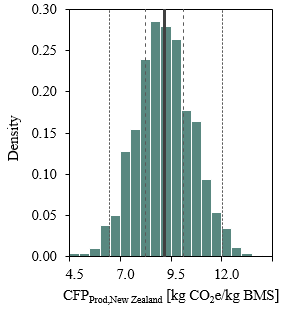

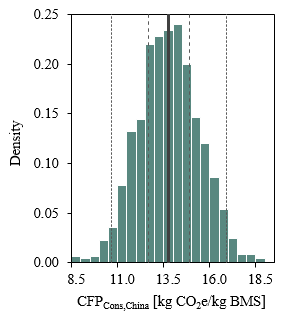


Figure S3. Monte-Carlo simulation of uncertainties in the production CFP for the New Zealand case (left) and the consumption CFP for the Chinese case (right). Dashed and dotted lines represent the 50% and 95% confidence intervals, respectively, and the solid line represents the sample median.


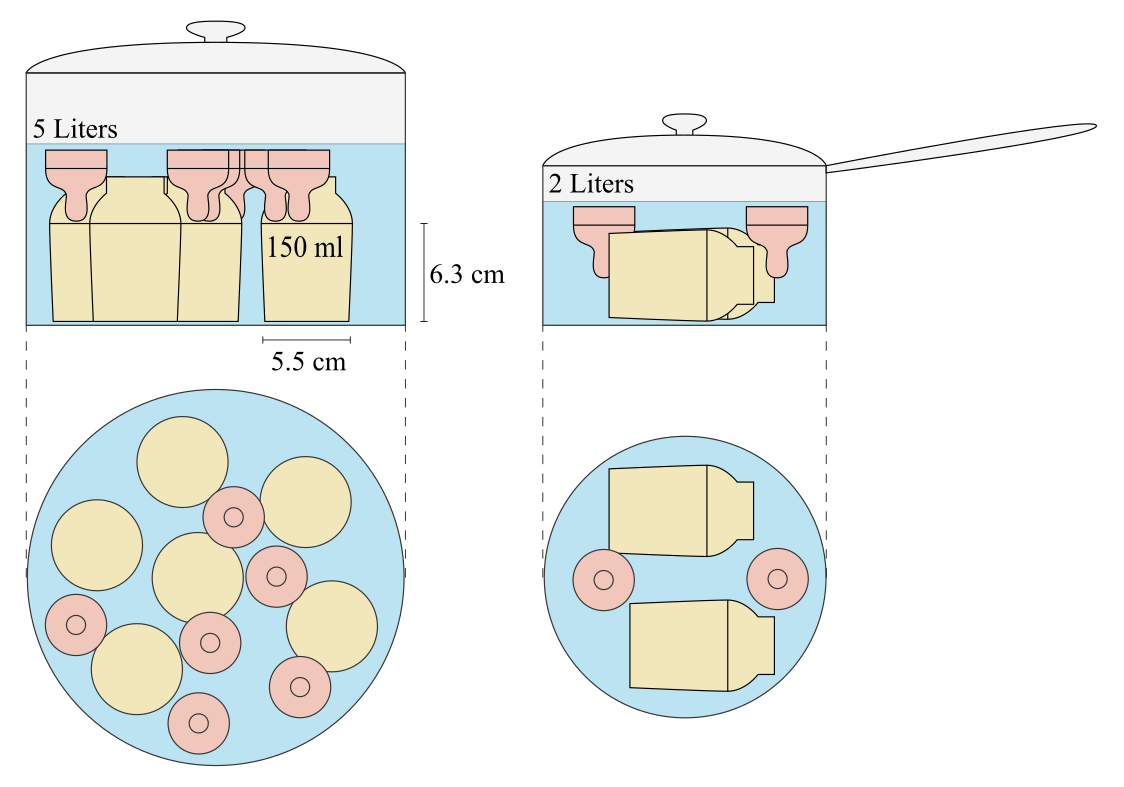


Figure S4. Illustration of number of 150 mL baby bottles sterilised at a time using the stove-top method.

# References

Brander, M., Sood, A., Wylie, C., Haughton, A., Lovell, J., 2011. Technical Paper | Electricity-specific emission factors for grid electricity. econometrica. <https://ecometrica.com/assets/Electricity-specific-emission-factors-for-grid-electricity.pdf> (Accessed 2018-10-26)

Clune, S., Crossin, E., Verghese, K., 2017. Systematic review of greenhouse gas emissions for different fresh food categories. Journal of Cleaner Production 140, 766-783. <https://doi.org/10.1016/j.jclepro.2016.04.082>

Dalgaard, R., Schmidt, J., Halberg, N., Christensen, P., Thrane, M., Pengue, W.A., 2007. LCA of soybean meal. The International Journal of Life Cycle Assessment 13(3), 240. <https://doi.org/10.1065/lca2007.06.342>

GEA, 2010. Milk Powder Technology - Evaporation and Spray Drying. <https://www.gea.com/en/binaries/Milk%20Powder%20Technology%20-%20Evaporation%20and%20Spray%20Drying_tcm11-33784.pdf> (Accessed 2018-10-26)

Gearghty, R., 2011. Resource Efficiency in Ireland’s Dairy Processing Sector - Benchmarking Resource Efficiency in Irish Dairy Processing. Enterprise Ireland. <https://www.leanbusinessireland.ie/includes/documents/Resource%20Efficiency%20Dairy%20Processing%20Ireland%20TOTAL%20JUNE%202011.pdf> (Accessed 2018-11-16)

Gustavsson, J., Cederberg, C., Sonesson, U., Van Otterdijk, R., Meybeck, A., 2011. Global food losses and food waste. FAO Rome. <http://www.fao.org/docrep/014/mb060e/mb060e00.pdf> (Accessed 2018-10-26)

Hagemann, M., Hemme, T., Ndambi, A., Alqaisi, O., Sultana, M.N., 2011. Benchmarking of greenhouse gas emissions of bovine milk production systems for 38 countries. Animal Feed Science and Technology 166-167, 46-58. <https://doi.org/10.1016/j.anifeedsci.2011.04.002>

Hager, T.J., Morawicki, R., 2013. Energy consumption during cooking in the residential sector of developed nations: A review. Food Policy 40, 54-63. <https://doi.org/10.1016/j.foodpol.2013.02.003>

Henders, S., Persson, U.M., Kastner, T., 2015. Trading forests: Land-use change and carbon emissions embodied in production and exports of forest-risk commodities. Environmental Research Letters 10(12). <https://doi.org/10.1088/1748-9326/10/12/125012>

Hui, Y.H., 2006. Handbook of Food Science, Technology, and Engineering. Taylor & Francis.

IPCC, 2006. 2006 IPCC Guidelines for National Greenhouse Gas Inventories. Prepared by the National Greenhouse Gas Inventories Programme.

Kažimírová, V., 2013. Heat Consumption and Quality of Milk Pasteurization. Acta Technologica Agriculturae 16(2), 55. <https://doi.org/10.2478/ata-2013-0014>

Rak, A., 2011. Energy Efficiency in Mechanical Separation. Water Arabia 2011 - Bahrain. <http://www.sawea.org/pdf/Andreas_Rak.pdf> (Accessed 2018-10-12)

Ramírez, C.A., Patel, M., Blok, K., 2006. From fluid milk to milk powder: Energy use and energy efficiency in the European dairy industry. Energy 31(12), 1984-2004. <https://doi.org/10.1016/j.energy.2005.10.014>

Röös, E., 2014. Mat-klimat-listan. Report / Department of Energy and Technology, SLU (1654-9406), 33. <https://pub.epsilon.slu.se/11671/> (Accessed 2018-09-25)

Schmidt, J.H., 2015. Life cycle assessment of five vegetable oils. Journal of Cleaner Production 87, 130-138. <https://doi.org/10.1016/j.jclepro.2014.10.011>

Smith, K., 2008. Dried Dairy Ingredients. Wisconsin Center for Dairy Research. <https://www.cdr.wisc.edu/sites/default/files/insider/resources/dried_dairy_ingdients.pdf> (Accessed 2018-09-11)

UK Department of Energy & Climate Change, 2013. Energy Follow-Up Survey 2011 - Report 9: Domestic appliances, cooking & cooling equipment. Department of Energy & Climate Change. <http://doc.ukdataservice.ac.uk/doc/7471/mrdoc/pdf/7471_9_domestic_appliances_cooking_and_cooling_equipment.pdf> (Accessed 2018-09-26)

Wallman, M., Nilsson, K., 2011. Klimatpåverkan och energianvändning från livsmedelsförpackningar [Climate impact and energy use from food packaging]. Livsmedelsverket. <https://www.livsmedelsverket.se/globalassets/publikationsdatabas/rapporter/2011/2011_livsmedelsverket_18_klimatpaverkan_energianvandning_livsmedelsforpackningar.pdf> (Accessed 2018-09-26)
